# Long-Term Reproducibility of Axial Length after Combined Phacovitrectomy in Macula-sparing Rhegmatogenous Retinal Detachment

**DOI:** 10.1038/s41598-018-34266-1

**Published:** 2018-10-26

**Authors:** Tae-Seen Kang, Hye-Jin Park, Young-Joon Jo, Jung-Yeul Kim

**Affiliations:** Department of Ophthalmology, Chungnam National University College of Medicine, Daejeon, Republic of Korea

## Abstract

There is a myopic shift in the final measured spherical equivalent following combined phacovitrectomy compared to the predicted postoperative value. This change in myopia is known to be associated with gas tamponade, but it also occurs in patients who do not have gas tamponade, and even when vitrectomy is performed in the pseudophakic eye. In this study, we focused on the long-term reproducibility of axial length after combined phacovitrectomy in patients with macula-sparing rhegmatogenous retinal detachment. Before surgery, one year after surgery, and two years after surgery, bilateral axial lengths were measured using partial interferometry. To confirm whether axial lengths changed after surgery, we conducted confidence analyses using the intraclass correlation coefficient (ICC), coefficient of variation (CV), and test–retest standard deviation (TRTSD). The preoperative mean axial length was 25.03 ± 1.69 mm in the affected eyes and 24.96 ± 1.70 mm in the fellow eyes. The ICC, CV, and TRTSD were 0.97, 0.45, and 0.114 in affected eyes and 0.98, 0.66, and 0.167 in fellow eyes, respectively, which shows a high level of reproducibility. Prediction errors for postoperative spherical equivalents measured using partial interferometry were −0.41 ± 0.67 diopters (p = 0.001), respectively, which shows a remarkable myopic shift. Correlation analyses indicated that this myopic shift was significant in eyes with a shallower anterior chamber and a thicker lens. In macula-sparing RRD patients, the axial length showed excellent long-term reproducibility two years after vitrectomy, cataract surgery, and gas tamponade. The myopic shift after surgery was therefore caused by factors that may have affected the intraocular lens position, such as preoperative anterior chamber depth and lens thickness, rather than a change in the axial length.

## Introduction

Rhegmatogenous retinal detachment (RRD) is an eye disorder that occurs when liquified vitreous humor flows into the potential space between the neurosensory retina and the underlying retinal pigment epithelium, resulting in separation of the neurosensory retina from the underlying retinal pigment epithelium. The two primary treatments for RRD involve scleral buckling and pars plana vitrectomy^1^. With recent developments in surgical devices and skills, there is a growing trend to perform vitrectomy with strong points that may directly eliminate traction and immediately remove subretinal fluid^2^. However, because vitrectomy produces a cataract^3^, secondary cataract surgery is then required. In recent years, combined phacoemulsification-vitrectomy (phacovitrectomy) has been performed more frequently because its safety has been confirmed^4^.

The postoperative spherical equivalent is an important factor that determines the satisfaction level of patients. There is a myopic shift in the final measured spherical equivalent following combined phacovitrectomy compared to the predicted postoperative value^5–10^. This change in myopia is known to be associated with gas tamponade^5^, but it also occurs in patients who do not have gas tamponade^11^, and even when vitrectomy is performed in the pseudophakic eye^10^. In previous studies, changes in axial length, as well as changes in the position of the intraocular lens, were known to be the cause of the myopic shift^11,12^. This change in axial length was explained by preoperative measurement errors^12–14^ or decreased macular edema^11^. Thus, it is important to measure these parameters precisely, even in combined phacovitrectomy.

However, numerous recent studies have reported limitations in axial length measurements for patients with macular edema such as epiretinal membrane, diabetic retinopathy, and macular hole^6–9^. It can be difficult to precisely measure this parameter because of unstable fixation and the accompanying decrease in visual acuity. In addition, errors could occur because of changes in the macula itself before and/or after surgery. In theory, such changes do not affect the axial length if the retinal pigment epithelium is preserved, because partial interferometry measures the distance from the corneal epithelium to the retinal pigment epithelium layer. However, there is a possibility of error if the macula is abnormal because of epiretinal membrane or macular edema^15^. When measuring patients with macula-off retinal detachment using partial interferometry, in theory, the axial length should not be altered because the pigment epithelium is in place. However, in practice, the axial length is underestimated by several millimeters.

We, therefore, aimed to determine whether combined phacovitrectomy might affect axial length measurements over an extended period. RRD patients with macula sparing were included. We measured the axial length three times at intervals of >1 year to determine possible long-term changes after combined phacovitrectomy. We then evaluated whether combined phacovitrectomy caused a myopic shift and identified factors affecting this shift by comparing the predicted spherical equivalent before surgery and the spherical equivalent measured two years after surgery.

## Methods

### Study design

We retrospectively analyzed the medical records of patients recruited from a single medical institution. The study protocol was approved by the institutional review board of Chungnam National University Hospital (Daejeon, Republic of Korea) and adhered to the tenets of the Declaration of Helsinki. The requirement for obtaining informed patient consent was waived due to the retrospective nature of the study. Vitrectomized eyes of patients fulfilling the inclusion criteria were included in the study group. Fellow eyes were included in the control group.

### Participants

Patients with macula-sparing RRD who were admitted to Chungnam University Hospital from January 2011 to December 2014 were included in this study. Patients underwent vitrectomy, cataract surgery, and gas tamponade. All patients received examinations at their first visit, including best-corrected visual acuity (BCVA), intraocular pressure (IOP), slit lamp biomicroscopy, axial length, optical coherence tomography, and fundus photography.

Inclusion criteria included macula-sparing RRD and a BCVA ≥ 0.7 (based on a Snellen chart) before surgery. Exclusion criteria were treatment with silicone oil tamponade during surgery, history of intraocular surgery, and retinal illness besides RRD (such as epiretinal membrane or retinal artery occlusion). Patients who had a preoperative macular thickness ≥300 µm; those whose axial length was measured using only partial interferometry; and those who had ocular media opacities, had macular epiretinal membrane, or developed cystoid macular edema after surgery were also excluded.

### Axial length measurements

Axial lengths were measured bilaterally before surgery, one year after surgery, and two years after surgery. These measured values were analyzed collectively. Before surgery, axial lengths were measured using partial interferometry (IOL Master^®^, Carl Zeiss, Jena, Germany) and remeasured using a contact ultrasound (Ocuscan RxP^®^, Alcon Laboratories, Fort Worth, TX, USA) 2 min later. After surgery, axial lengths were only measured using partial interferometry. We analyzed the confidence of measurements by comparing the measured results of vitrectomized eyes and fellow eyes.

The IOL power was directed toward emmetropia, which was calculated using the SRK/T formula. The calculation was performed separately using partial interferometry and ultrasound. If the IOL powers measured using the two methods varied by ≥1 diopter (D), the axial lengths were <22 mm or >25 mm, or the bilateral axial lengths differed from each other by ≥0.33 mm, the measurements were repeated by other examiners, and then the results were confirmed.

### Surgical methods

Combined 23-gauge sutureless phacovitrectomies were performed under retrobulbar anesthesia by a single surgeon (J.Y.K.). Cataract extraction preceded pars plana vitrectomy after the insertion of a trocar. A 2.8 mm clear corneal incision was made with a superior approach, and the anterior chamber was filled with a viscoelastic substance (1.4% sodium hyaluronate). A 4.5 mm diameter CCC of the anterior capsule was performed followed by cortical cleaving hydrodissection. A standard phacoemulsification was performed, and the residual cortex was removed by irrigation/aspiration. A vitrectomy was performed to repair the RRD after the anterior chamber was filled with a viscoelastic substance. The Constellation Vision System^®^ (Alcon Laboratories) was used for vitrectomies. The subretinal fluid was discharged using a fluid-air exchange after perfluorocarbon liquids were administered. Endolaser photocoagulation was performed around the breaks. Then a three-piece spherical acrylic IOL with a 6.0 mm optical zone diameter produced by Sensar^®^ (AR40e; Abbot Medical Optics, Santa Ana, CA, USA) was inserted into the posterior capsule just before the SF_6_ or C_3_F_8_ gas tamponade. An incision suture was not made, and there was no leakage after surgery.

### Statistical analyses

SPSS for Windows, version 18.0 (SPSS, Chicago, IL, USA), was used for all statistical analyses. The long-term reproducibility was determined using the intraclass correlation coefficient (ICC), coefficient of variation (CV), and test–retest standard deviation (TRTSD). The TRTSD was calculated as follows. Axial lengths were measured three times were averaged, and the standard deviation of the mean value of each measured value was defined as the TRTSD. We calculated the ICC by dividing the within-subject variance by the total variance. We calculated the CV by dividing the TRTSD by the mean of total measured values, then multiplying by 100. A *p-*value < 0.05 was considered statistically significant.

## Results

### Participants

A total of 288 patients were diagnosed with RRD from January 2011 to December 2014, and 101 of these patients were treated with combined phacovitrectomy. Of these patients, 77 patients were followed up for >2 years after surgery. A total of 40 patients were excluded, including those whose axial lengths were difficult to measure because of preoperative vitreous hemorrhage (*n* = 15), those with macula-off RRD (*n* = 23), and those who had macular edema (*n* = 1) or epiretinal membrane after surgery (*n* = 1). Ultimately a total of 37 patients were analyzed in this study: 20 males and 17 females with a mean age of 56.5 ± 6.2 years. Their mean postoperative follow-up period was 25.9 months. Of these patients, one had been diagnosed with diabetes, and five had had hypertension as a systemic disease before surgery. All patient symptoms were well controlled by oral medications. No other systemic diseases were found. RRD developed in the right eyes of 22 patients and the left eyes of 15 patients. Retinal tear, retinal hole, and both retinal tear and retinal hole caused RRD in 29, seven, and one patient(s), respectively. For the fluid-air exchange, C_3_F_8_ gas was used in 32 patients, whereas SF_6_ gas was used in five patients. Proliferative vitreoretinopathy was not detected in any patient (Table 1).

**Table 1:** Demographic characteristics and preoperative findings.

Age (years, mean ± SD)	56.5 ± 6.2
**Sex**, ***n*** **(%)**	
Male	20 (54.1)
Right laterality (%)	22 (59.5)
Type of break (Tear/hole, *n*)	30/7
Number of breaks (*n* ± SD)	1.43 ± 0.73
Single break (*n*, %)	17 (45.9)
Multiple breaks (*n*, %)	20 (54.1)
**Position of break**	
Superior (*n*, %)	25 (67.6)
Inferior (*n*, %)	6 (16.2)
Both superior and inferior (*n*, %)	6 (16.2)
Gas tamponade use	
C_3_F_8_ (*n*, %)	32 (86.5)
SF_6_ (*n*, %)	5 (13.5)

The preoperative spherical equivalents of affected and fellow eyes were −2.7 ± 3.6 D and −2.8 ± 3.0 D, respectively (*p* = 0.756). During follow-up, three patients underwent phacoemulsification in the fellow eye to correct anisometropia. The bilateral IOP (*p* = 0.668) and corneal spherical equivalent (*p* = 0.366) were within normal ranges and were not significantly different between affected and fellow eyes (Table 3).

### Reproducibility of axial length measurements

In all patients, axial length measurements were performed using partial interferometry and ultrasound prior to combined phacovitrectomy. The measurements were repeated in 29 patients. There were twenty-two patients whose axial lengths were longer than 25 mm, or whose difference of binocular axial length was more than 0.33 mm. These re-measurements did not affect the decisions of IOL. In seven patients (18.9%), the difference between ultrasound and partial interferometry was more than 0.33 mm, and IOL was decided by an additional test. The preoperative axial lengths of affected eyes and fellow eyes using partial interferometry were 25.4 ± 1.67 mm and 25.2 ± 1.69 mm, respectively. Using ultrasound, they were 25.2 ± 1.61 mm and 25.0 ± 1.65 mm, respectively. The axial lengths using partial interferometry were approximately 0.15 mm longer than those using ultrasound. This difference was statistically significant (*p* < 0.001). Whereas ultrasound measured the distance from the corneal peak to the internal limiting membrane, partial interferometry measured the distance from the corneal peak to the retinal pigment epithelium. Therefore, partial interferometry might have measured a longer axial length because of the retinal thickness of the macula.

**Figure 1:**
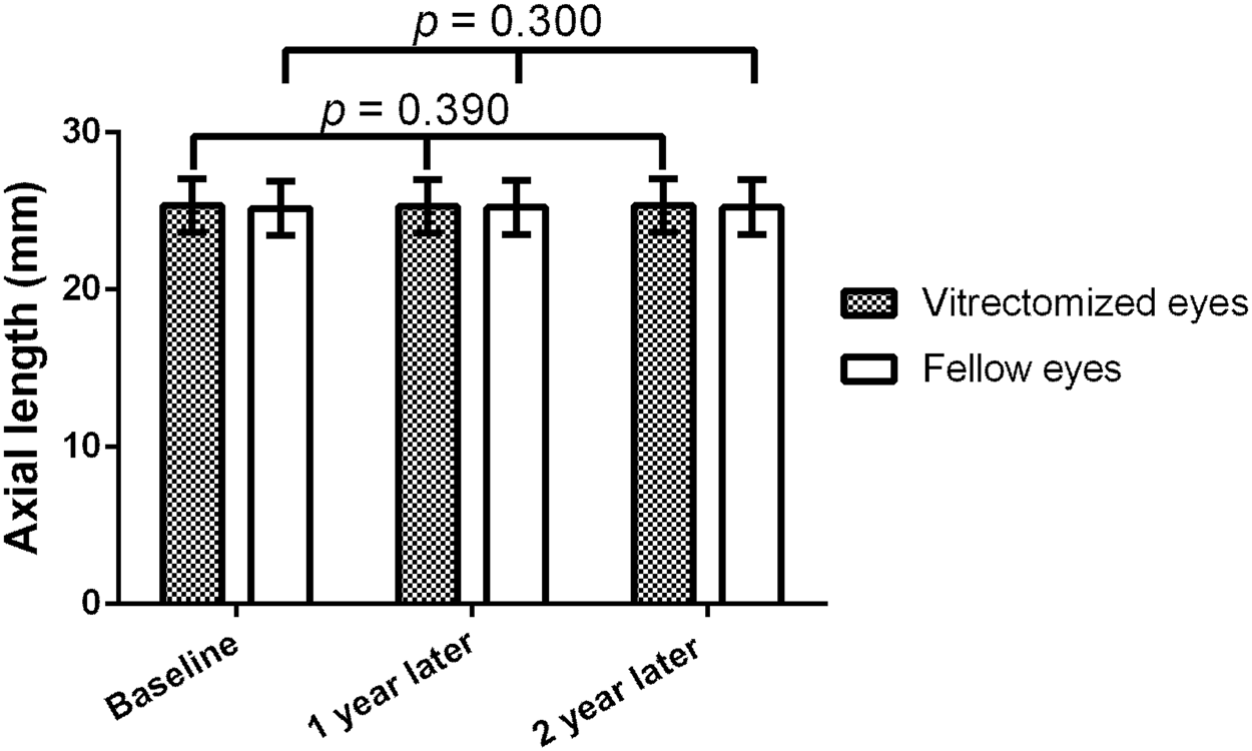
Axial length changes in vitrectomized and fellow eyes. The one-way analysis of variance (ANOVA) is used to determine whether the means of axial lengths are unchanged. The axial lengths were measured by partial interferometry.

**Figure 2:**
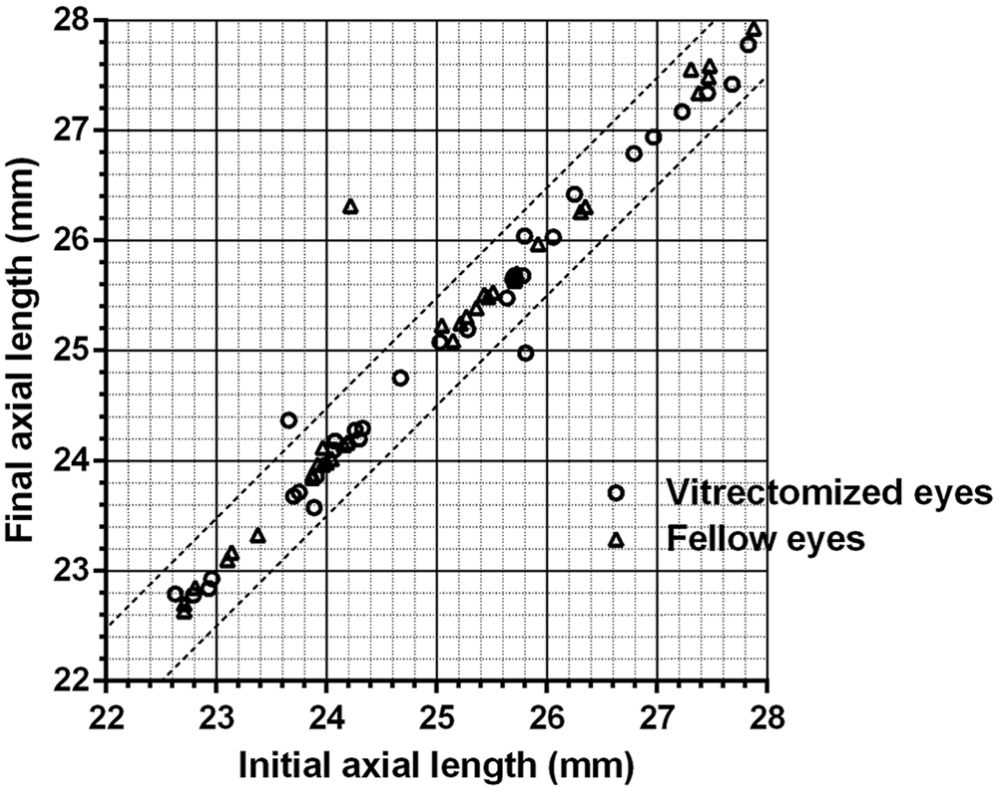
Initial and final axial lengths using partial interferometry in all patients. Circles and triangles between the two lines denote changes in the axial length ≤0.5 mm. Circles and triangles outside of the two lines denote changes >0.5 mm.

One year and 2 years after surgery, the axial lengths measured using partial interferometry were 25.3 ± 1.68 mm and 25.3 ± 1.78 mm for affected eyes, respectively. According to repeated measures analysis of variance (ANOVA), they were not significantly different from the preoperative axial length (25.4 ± 1.67 mm). The axial lengths of fellow eyes 1 year and 2 years after surgery were 25.2 ± 1.70 mm and 25.2 ± 1.71 mm, respectively. They were also not significantly different from the preoperative axial length (25.2 ± 1.69 mm; Fig. 1). Except for three cases, the axial length changed to within 0.5 mm (Fig. 2).

We analyzed the confidence of axial length measurements using the ICC, CV, and TRTSD. For affected and fellow eyes, the ICCs were 0.97 and 0.98, the CVs were 0.45% and 0.66%, and the TRTSDs were 0.114 and 0.167, respectively. These results showed remarkable reproducibility in measurements for both affected and fellow eyes (Table 2). High reproducibility was also detected after we measured the axial lengths using partial interferometry in macula-sparing RRD patients following combined phacovitrectomy. There was no significant change in the axial lengths.

**Table 2:** The intraclass correlation coefficient (ICC), the coefficient of variation (CV), and test–retest standard deviation (TRTSD) of the axial lengths.

	ICC^*^	CV, %^†^	TRTSD^‡^
Vitrectomized eyes	0.970 (0.945–0.984)	0.45	0.114
Fellow eyes	0.980 (0.963–0.990)	0.66	0.167

^*^Data are presented as ICCs with the 95% confidence intervals in parentheses.

^†^Calculated as 100 × within-subject standard deviation/average of measurements.

^‡^Calculated as the square root of the within-subject variance.

### Visual acuity

BCVA was calculated based on a logMAR chart. At the first examination, the mean BCVA of affected eyes was 0.18 ± 0.28, and that of fellow eyes was 0.05 ± 0.50. One year and 2 years after surgery, the mean BCVA values of affected eyes were 0.08 ± 0.18 and 0.13 ± 0.32, respectively. Those of fellow eyes were 0.08 ± 0.30 and 0.10 ± 0.28, respectively. The mean BCVA of the fellow eyes was significantly (*p* = 0.034) better than that of affected eyes before surgery. After surgery, there were no significant differences in BCVA values between affected and fellow eyes (1 year later, *p* = 0.843; 2 years later, *p* = 0.641). Repeated measures ANOVA also found no significant difference in BCVA values for affected eyes and fellow eyes (affected eyes, *p* = 0.339; fellow eyes, *p* = 0.765).

### Corneal curvature

The corneal curvature was measured using partial interferometry At the first examination, the corneal curvature of vitrectomized eyes and fellow eyes was 43.3 ± 1.94 and 43.2 ± 1.90, respectively. One year or 2 years after surgery, there were no significant differences in the corneal curvature between affected and fellow eyes (Table 3).

**Table 3:** Best-corrected visual acuity, spherical equivalent, and intraocular pressure in vitrectomized and fellow eyes.

	Baseline characteristics	Postoperative 1 Year	Postoperative 2 Years	*p*-value
**Vitrectomized eyes**				
BCVA (logMAR, mean ± SD)	0.14 ± 0.17	0.05 ± 0.10	0.08 ± 0.20	0.119
SE (D, mean ± SD)	−2.5 ± 3.6	−1.1 ± 1.0	−1.1 ± 2.1	0.125
IOP (mmHg, mean ± SD)	16.0 ± 2.6	16.1 ± 3.5	16.0 ± 3.1	0.984
Keratometry (D ± SD)	43.3 ± 1.94	43.3 ± 2.07	43.2 ± 2.01	0.988
**Fellow eyes**				
BCVA (Log MAR, mean ± SD)	0.01 ± 0.04	0.01 ± 0.02	0.04 ± 0.16	0.350
SE (D, mean ± SD)	−2.5 ± 3.0	−1.7 ± 2.4	−1.7 ± 3.3	0.515
IOP (mmHg, mean ± SD)	16.1 ± 3.4	15.7 ± 3.2	15.7 ± 2.8	0.821
Keratometry (D ± SD)	43.2 ± 1.90	42.9 ± 2.20	43.0 ± 2.18	0.843

BCVA, best corrected visual acuity; logMAR, the logarithm of the minimum angle of resolution; SE, spherical equivalent; D, diopters; IOP, intraocular pressure.

^*^Analysis of variance.

### The myopic shift in postoperative refraction

**Figure 3:**
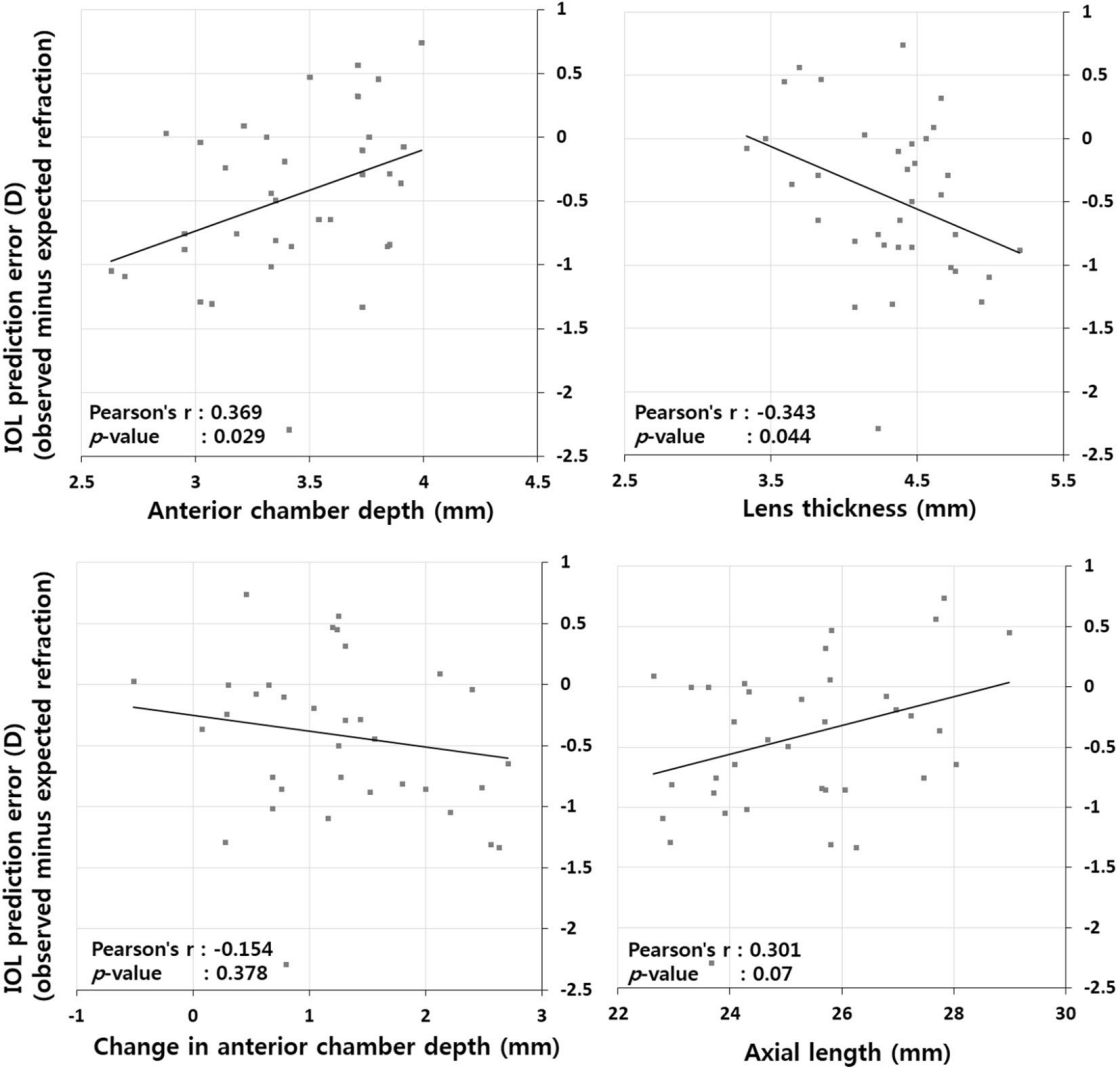
Correlation analyses of relationships between preoperative factors and intraocular lens prediction errors (observed minus expected refraction). A shallower anterior chamber and thicker lens resulted in higher myopic shifts.

The predicted spherical equivalent before surgery was compared to that 2 years after surgery. The spherical equivalent error values using partial interferometry and ultrasound were −0.41 ± 0.67 D (*p* = 0.001) and −0.49 ± 0.68 D (*p* < 0.001), respectively, which indicates a significant myopic shift. Although the spherical equivalent error using partial interferometry was slightly smaller than that using ultrasound, the difference was not statistically significant (*p* = 0.623). To determine the causes of this myopic shift, we performed correlation analyses of axial lengths using partial interferometry, anterior chamber depths (ACDs) using partial interferometry, changes between the perioperative ACDs, ACDs using ultrasound, and lens thickness using ultrasound. When the ACD using ultrasound was shallower (*p* = 0.029) and the lens thickness using ultrasound was thicker (*p* = 0.044), a myopic shift was apparent. Other factors did not significantly affect this correlation (Fig. 3). Preoperative ACDs lens thickness, axial length were regression analyzed. The ACD and the thickness of the lens showed significant results (Table 4).

**Table 4:** Univariate linear regression analysis of factors affecting intraocular lens prediction error.

	coefficient	R^2^	*p*-value
ACD	0.638	0.136	0.029
Lens thickness	−0.491	0.118	0.044
Axial length	0.119	0.091	0.070
Perioperative ACD change	−0.130	0.024	0.378
Lens thickness/Axial length	−7.054	0.448	0.002

ACD: Anterior chamber depth.

To examine whether the IOL power was affected by the type of gas used during surgery, we compared the change in the spherical equivalent between 32 patients treated with C_3_F_8_ and five patients treated with SF_6_. The IOL powers in the C_3_F_8_-administered group and SF_6_-administered group showed a myopic shift of −0.40 ± 0.68 D and −0.48 ± 0.71 D, respectively. The difference in the myopic shift between the two groups was not statistically significant (*p* = 0.87).

## Discussion

To the best of our knowledge, this is the first study to report how the axial length is changed over an extended period of time after combined phacovitrectomy in patients with macula-sparing RRD using partial interferometry. Prior studies have reported that partial interferometry has a high level of reproducibility^16^. However, whether partial interferometry has a high level of reproducibility for patients treated with vitrectomy has not yet been determined.

The spherical equivalent undergoes a myopic shift compared to the predicted spherical equivalent following combined phacovitrectomy. Rahman *et al*. investigated differences in the IOL power before and after combined phacovitrectomy in 95 RRD patients and reported that the IOL powers of the macula-on of 41 eyes and the macula-off of 54 eyes underwent a myopic shift of −0.23 D and −0.42 D, respectively, compared to predicted values^9^. Ehmann *et al*.^6^ and Schweitzer *et al*.^5^ also reported that the spherical equivalent underwent a myopic shift of −0.76 D and −0.46 D, respectively, in patients with macular hole. In addition, the spherical equivalent can undergo a myopic shift of approximately −0.13 D because of the influence of the vitrectomy, resulting from differences between the refractive indices of the vitreous body (1.3346) and aqueous humor (1.3336)^11^. However, these myopic shifts cannot be explained only by vitrectomy, which makes it difficult to determine the IOL power. To minimize this error, some surgeons have performed cataract surgery separately at a specific time after vitrectomy. However, this protocol requires a long recovery time with additional expenses. In addition, it increases the difficulty of the surgery because of the absence of a vitreous support.

The corneal curvature is not changed after 23-gauge sutureless vitrectomy. Schweitzer examined astigmatism induced after sutureless vitrectomy in 57 patients and reported that the corneal curvature was changed by 0.01 D^17^. In the present study, there were no significant differences in the corneal curvature 2 years after surgery. The preoperative corneal curvature did not contribute to changes in the spherical equivalent because it was stable over time. Thus, it was not a source of error after combined phacovitrectomy and IOL implantation.

The axial length status after vitrectomy is therefore important in determining the IOL power. In a study using partial interferometry, Huang *et al*. reported that the axial length was elongated soon after scleral buckling. However, it was not significantly different 12 months after vitrectomy^18^. Nonetheless, different results were observed in studies that measured the axial length using ultrasound. Jeoung *et al*. reported that the axial length was significantly increased in patients with an axial length ≥24.5 mm 4 months after combined phacovitrectomy involving 154 eyes with diverse illnesses^19^. Kovacs *et al*. also reported that the spherical equivalent underwent a myopic shift of −0.79 D after combined phacovitrectomy in 12 epiretinal membrane patients compared to predicted values using ultrasound. The axial length was also increased with a reduction of macular edema after surgery^11^. Taken together, these results indicate that axial length measurement methods and macular lesions may affect axial length measurements.

Therefore, further study of patients with a normal macula who undergo combined phacovitrectomy is needed. In this study, surgery was performed only on macula-sparing RRD patients; the maculae were thus not affected after surgery. Also, we excluded patients with other retinal diseases that might have affected the macula.

As expected, the present study showed a myopic shift that was observed using either partial interferometry or ultrasound. However, no significant differences were found between the two methods. Correlation analyses were performed to identify possible causes of this myopic shift, but there was no significant correlation with age, sex, axial length, corneal curvature, changes in perioperative ACD, or type of gas tamponade. However, a myopic shift of the spherical equivalent correlated moderately with a thicker preoperative lens and a shallower ACD.

The SRK/T formula was used to determine the preoperative IOL power in a variety of studies involving myopic shifts of the IOL power after combined phacovitrectomy^5,6,9,10^. Using this formula, an effective lens position (ELP) has also been shown to improve the accuracy. The ELP is the distance from the corneal peak to the IOL. It is divided into two parts: from the corneal peak to the iris and from the iris to the IOL. The distance from the corneal peak to the iris is calculated mathematically using the axial length and corneal curvature, which is used to calculate the IOL power. In this study, neither axial length nor corneal curvature affected the postoperative change in the IOL power, because there were no changes in these parameters over time. Based on the SRK/T formula, the distance from the iris to the IOL was assumed to be constant using a type of IOL referred to as “offset”. This suggests that the myopic shift of the IOL power after combined phacovitrectomy in RRD patients was due to the fact that the IOL was located within a shorter distance than the assumed offset (i.e., closer to the iris). In addition, the myopic shift of the IOL power was preferentially found in eyes with a shallower anterior chamber and thicker lens.

This might be because of limitations in the SRK/T formula, which only uses the corneal curvature and axial length to estimate the anterior chamber location after surgery. Thus, another formula capable of estimating the postoperative ACD using the preoperative ACD and lens thickness is needed^20^. The IOL is inserted to lens capsule in the cataract surgery. If the volume of the lens is large, the IOL can be positioned more freely. Therefore, it is reasonable to assume that the distance between the IOL and the cornea may differ from the expected value when the lens is thick. Also, the thicker the lens, the shallower the anterior chamber depth(r = −0.597, *p* < 0.001), so it can be explained that the shallower the anterior chamber depth, the higher the error.

A possible explanation for the offset change is that the IOL may have drifted toward the anterior chamber because of gas pressure after a fluid-air exchange. Within the vitreous cavity, C_3_F_8_ is known to be maintained for 6–8 weeks, and SF_6_ is sustained for 1–2 weeks. David *et al*. suggested that the IOL power can undergo a myopic shift when the IOL is pushed to the anterior chamber after a fluid-air exchange^10^. This change can be caused by a number of risk factors, including sulcus IOL implantation, large continuous anterior capsulotomy, and a flexible IOL^10^. Suzuki *et al*.^21^ and Sharma *et al*.^22^ also reported a myopic shift after vitrectomy and gas tamponade. Moreover, Hwang *et al*. reported that the extent of changes in the ACD and myopia varied in patients with macular hole depending on the nature of the IOL^7^. The gas used in those studies could also have affected the IOL power, because the rate of expansion and persistency depended on the type of gas. Byrne *et al*. reported that the use of C_3_F_8_ (which has a longer persistence than SF_6_) led to an increased myopic shift^10^. Kim *et al*. reported that the myopic shift in both phakic eyes and IOLs was greater in patients with macular hole after the use of C_3_F_8_ compared to SF_6_^8^. Given the myopic shift from long-lasting gas, gas tamponade could affect the healing process such as capsular fibrosis, and it might change the IOL position permanently. However, in this study, there were no significant differences between the two gases used, which might be because SF_6_ was used in only five patients.

Another explanation is that the vitreous and lens surface interaction disappears after the vitrectomy, and it induces the IOL shift. The vitreous and lens are not entirely independent tissues. Weigert’s ligament which is a condensed vitreous on the lens surface and Cloquet’s canal formed by degeneration of the primary vitreous adheres to the back surface of the lens. Therefore, we think that vitrectomy could affect the posterior surface of the lens, and it may affect the IOL position. The gas tamponade might make this interaction more apparent through physical properties of the gas such as surface tension.

A limitation of this study is that both vitrectomy and gas tamponade were performed in patients with macula-sparing RRD. To determine whether vitrectomy per se affects the axial length, vitrectomy should be performed in the absence of fluid-gas exchange in patients with normal maculae. However, it is difficult to find indications for such surgery. Further studies should be conducted in patients treated via vitrectomy alone.

In conclusion, axial lengths were not altered in patients with macula-sparing RRD 2 years after vitrectomy, cataract surgery, and gas tamponade. High reproducibility in axial length measurements was found in affected and fellow eyes. The preoperative ACD and lens thickness may have affected the postoperative location of the IOL, rather than any change in the axial length, inducing a postoperative myopic shift.

## Data Availability

Data supporting the findings of the current study are available from the corresponding author on reasonable request.
